# Comparative Genomics of *Thalassobius* Including the Description of *Thalassobius activus* sp. nov., and *Thalassobius autumnalis* sp. nov.

**DOI:** 10.3389/fmicb.2017.02645

**Published:** 2018-01-12

**Authors:** María J. Pujalte, Teresa Lucena, Lidia Rodrigo-Torres, David R. Arahal

**Affiliations:** Departamento de Microbiología and Colección Española de Cultivos Tipo, Universitat de València, Valencia, Spain

**Keywords:** *Thalassobius*, taxogenomics, phylogenomics, *Rhodobacteraceae*, *Roseobacter* group, *Shimia*, *Thalassococcus*

## Abstract

A taxogenomic study was conducted to describe two new *Thalassobius* species and to analyze the internal consistency of the genus *Thalassobius* along with *Shimia* and *Thalassococcus*. Strains CECT 5113^T^, CECT 5114, CECT 5118^T^, and CECT 5120 were isolated from coastal Mediterranean seawater, Spain. Cells were Gram-negative, non- motile coccobacilli, aerobic chemoorganotrophs, with an optimum temperature of 26°C and salinity of 3.5–5%. Major cellular fatty acids of strains CECT 5113^T^ and CECT 5114 were C_18 : 1_ ω7c/ω6c and C_10 : 0_ 3OH, G+C content was 54.4–54.5 mol% and were able to utilize propionate, L-threonine, L- arginine, and L-aspartate as carbon sources. They exhibited 98.3% 16S rRNA gene sequence similarity, 75.0–75.1 ANIb and 19.5–20.9 digital DDH to type strain of their closest species, *Thalassobius maritimus*. Based on these data, strains CECT 5113^T^ and CECT 5114 are recognized as a new species, for which the name *Thalassobius activus* is proposed, with strain CECT 5113^T^ (=LMG 29900^T^) as type strain. Strains CECT 5118^T^ and CECT 5120 were found to constitute another new species, with major cellular fatty acids C_18 : 1_ ω*7c*/ω*6c* and C_18 : 1_ ω*7c* 11-methyl and a G+C content of 59.8 mol%; they were not able to utilize propionate, L-threonine, L- arginine or L-aspartate. Their closest species was *Thalassobius mediterraneus*, with values of 99.6% 16S rRNA gene sequence similarity, 79.1% ANIb and 23.2% digital DDH compared to the type strain, CECT 5383^T^. The name *Thalassobius autumnalis* is proposed for this second new species, with strain CECT 5118^T^ (=LMG 29904^T^) as type strain. To better determine the phylogenetic relationship of the two new species, we submitted 12 genomes representing species of *Thalassobius, Shimia*, and *Thalassoccocus*, to a phylogenomic analysis based on 54 single protein-encoding genes (BCG54). The resulting phylogenomic tree did not agree with the current genera classification, as *Thalassobius* was divided in three clades, *Thalassobius* sensu stricto (*T. mediterraneus, T. autumnalis* sp. nov., and *T. gelatinovorus*), *Thalassobius aestuarii* plus the three *Shimia* spp (*S. marina, S. haliotis*, and *Shimia* sp. SK013) and finally, *Thalasobius maritimus* plus *T. activus* sp. nov. *Thalassococcus halodurans* remained apart from the two genera. Phenotypic inferences from explored genomes are presented.

## Introduction

The genus *Thalassobius* was established to accommodate the species *Thalassobius mediterraneus* and the reclassified *Thalassobius gelatinovorus* (formerly *Ruegeria gelatinovorans*) by Arahal et al. (2005). It is affiliated to the *Roseobacter* group, in the family *Rhodobacteraceae*, class *Alphaproteobacteria* (Pujalte et al., 2014). Since the description of the first pair of species, five more have been added: *Thalassobius aestuarii* (Yi and Chun, 2006), *Thalassobius maritimus* (Park et al., 2012), *Thalassobius aquaeponti* (Park et al., 2014), *Thalassobius abyssi* (Nogi et al., 2016), and *Thalassobius litorarius* (Park et al., 2016). All species so far characterized are aerobic chemoorganotrophic marine bacteria able to accumulate polyhydroxybutyrate (PHB). They have been isolated from marine environments, particularly surface coastal seawater and tidal flat samples, but one species (*T. abyssi*) was isolated from deep seawater (around 1,000 m depth). Strains identified as *Thalassobius* sp. on the basis of 16S rRNA gene sequence have been isolated and reported from diverse marine samples, including corals, mollusks, sand, microalgal cultures, and mariculture samples of different types, and show a wide geographic distribution (from temperate zones such as Kuwait coast to polar regions, as Antarctica or Norwegian subartic fjords). Most species show complex ionic requirements, as they require seawater based media and are unable to grow in media containing only NaCl or just a combination of NaCl with calcium, magnesium or potassium salts. None of the species produces *BChl a* or synthetizes carotenoid pigments.

The *Roseobacter* group, which comprises a very large number of genera (100 at the time of the writing), expands at a quick rate. The phylogenetic position of the genus *Thalassobius* in the group has been addressed with 16S rRNA gene sequence comparisons: *Thalassobius* species form a clade in the phylogenetic trees, but it frequently incorporates sequences of *Thalassococcus* (LTP128, https://www.arb-silva.de/projects/living-tree/) or *Shimia* species (Pujalte et al., 2014). *Shimia* includes five species, *Shimia marina* (Choi and Cho, 2006), *Shimia isoporae* (Chen et al., 2011), *Shimia haliotis* (Hyun et al., 2013), *Shimia biformata* (Hameed et al., 2013), and *Shimia sagamensis* (Nogi et al., 2015) while *Thalassococcus* includes two, *Thalassococcus halodurans* (Lee et al., 2007) and *Thalassococcus lentus* (Park et al., 2013). The relationships of *Thalassobius* to both genera (*Shimia* and *Thalassococcus*) are thus unclear. The use of whole genome sequences and multiple gene trees would surely add confidence and resolution to the inference of their evolutionary relationships but this approach is, at present, limited by the gap in whole genome sequences from type strains. Unfortunately, recent phylogenomic studies on the *Roseobacter* group or the *Rhodobacteraceae* family have not included any reference genome of the genus *Thalassobius* (Newton et al., 2010; Tang et al., 2010; Luo and Moran, 2014; Simon et al., 2017).

Whole genome drafts of *T. mediterraneus* and *T. gelatinovorus* and *S. marina* type strains have been determined and characterized in the last two years (Rodrigo-Torres et al., 2016a,b, 2017). Also, Nogales and co-workers have explored the phylogenomics of a large collection of the *Roseobacter* group members, including several type strain genomes, by using more than a 100 single copy protein coding genes and found four *Thalassobius* species forming a well-defined lineage, which includes *Shimia* species (unpublished, Nogales et al., 2017, FEMS Meeting of European Microbiologists). Thus, the possible polyphyletic/paraphyletic nature of the genus is an open question.

During a survey aimed to resolve the taxonomic position of unidentified marine strains kept at the Spanish Type Culture Collection (CECT), whole genome sequences were obtained for four *Thalassobius* spp. strains. In this paper, we address the genome-based taxonomy of the genus *Thalassobius*, describe two new species of *Thalassobius* revealed through genome relatedness and present some predicted phenotypic traits of the group.

## Materials and methods

### Bacterial strains

The strains used in this study are listed in Table 1 with indication of their origins and references of previous studies. Marine Agar (MA) and Marine Broth (MB) were used as routine cultivation media and incubations were done at 26°C.

**Table 1:** Strains used in the study and their origins.

**Strain**	**Equivalent designation**	**Isolation**	**Reference**
*T. activus* CECT 5113^T^	11SM13^T^; LMG 29900^T^	Sea water, Vinaroz, Spain. November 1989	Phenon 21, (Ortigosa et al., 1994)
*T. activus* CECT 5114	11SM18; LMG 29901	Sea water, Vinaroz, Spain November 1989	Phenon 21, (Ortigosa et al., 1994)
*T. aestuarii* CECT 8650^T^	JC2049^T^; KCTC 12049^T^	Tidal flat sediment Ganghwa Island, Korea	Yi and Chun, 2006
*T. autumnalis* CECT 5118^T^	XSM11^T^; LMG 29904^T^	Sea water, Vinaroz, Spain October 1989	Phenon 34, (Ortigosa et al., 1994)
*T. autumnalis* CECT 5120	11SM11; LMG 29905	Sea water, Vinaroz, Spain November 1989	Phenon 35, (Ortigosa et al., 1994)
*T. gelatinovorus* CECT 4357^T^	Ahrens B6^T^; LGM 129^T^	Sediment, seawater, Kiel Fjord, Baltic Sea, Germany	Rüger and Höfle, 1992; Uchino et al., 1998
*T. maritimus* CECT 8648^T^	GSW-M6^T^; KCTC 23347^T^	Seawater, Geoje Island, Korea	Park et al., 2012
*T. mediterraneus* CECT 5383^T^	XSM19^T^; CIP 108400^T^	Seawater, Vinaroz, Spain October 1989	Arahal et al., 2005

### 16S rRNA gene sequence analysis

The complete 16S rRNA gene sequence of strains CECT 5113^T^, CECT 5114, CECT 5118^T^, and CECT 5120 were 1,465, 1,465, 1,469, and 1,469 nucleotides in length, respectively. These sequences extracted from the genome were compared with corresponding sequences of the type strains within the *Roseobacter* group using alignments retrieved from SILVA and LTP (Yarza et al., 2010) latest updates as references. When necessary, additional sequences were retrieved from the GenBank/EMBL/DDBJ databases. Alignments were corrected manually based on secondary structure information. Sequence similarities were calculated in ARB based on sequence similarities without the use of an evolutionary substitution model. Phylogenetic analysis using alternative treeing methods (maximum-parsimony, maximum-likelihood, and distance matrix) and data subsets were performed using the appropriate ARB tools (Ludwig et al., 2004).

### Whole genome sequencing and comparison

Genomic DNA was isolated using Real Pure Spin kit (Durviz) following the standard protocol recommended by the manufacturer. The integrity of the extracted DNA was checked by visualization in a 2.0% (w/v) agarose gel electrophoresis. Its purity and quantity was checked by measuring the absorbance at 260 and 280 nm with a spectrophotometer Nanodrop2000c (Thermo Scientific) and calculating the ratio A260/A280. Genome sequencing was achieved at Central Service of Support to Experimental Research (SCSIE) of the University of Valencia (Valencia, Spain) using an Illumina Miseq technology with 2 × 250 paired-end reads. The Illumina reads were analyzed for quality control using FASTQC, a common quality control tool developed by Babraham Bioinformatics to check raw sequencing data, which is wrapped in Galaxy Orione Server (Cuccuru et al., 2014). After filtering, the remaining reads were assembled using several software choices for comparative purposes: (i) Spades 3.0.0 (Bankevich et al., 2012) incorporated as a tool in Galaxy Orione Server, (ii) Seqman Ngen 12.0.1 (DNAstar), and (iii) Velvet 1.0.0 *de novo* assembler (Zerbino and Birney, 2008). After performance evaluation and comparison of metrics, the best assembly for each organism was further processed. The bioinformatic tool CheckM (Parks et al., 2015) was used to assess the genome quality prior to annotation using Prokka v1.4.0 (Seemann, 2014), an open source software tool within Galaxy Orione Server, and RAST v2.0 (Rapid Annotation using Subsystem Technology; Aziz et al., 2008).

The similarity between genomes was assessed using several indices useful for species delineation. Thus, the DNA-DNA hybridization (DDH) was estimated *in silico* with the Genome-to-Genome Distance Calculator (GGDC 2.0) using the BLAST method and recommended formula 2 (Meier-Kolthoff et al., 2013); the average nucleotide identities according to MUMmer (ANIm) and BLAST (ANIb) were determined in JSpeciesWS (Richter et al., 2015); and OrthoANI values were calculated with the standalone Orthologous Average Nucleotide Identity Tool (OAT) (Lee et al., 2016).

The phylogenetic relationship of the genomes was explored with BCG54 using default settings. This software tool is available for download at EzBioCloud (Yoon et al., 2017) and employs a set of bacterial core genes, namely 54, that are single-copy and commonly present in all bacterial genomes.

### Phenotypic characterization

All determinations were performed in duplicate in non-simultaneous assays. Cell morphology was determined on wet mounts prepared from 24 to 48 h MA cultures of the strains, by using phase contrast microscopy in a Leica DMRB fluorescence microscopy. Colony morphology and pigmentation were recorded from 48 h MA cultures. PHB accumulation was determined according to Spiekermann et al. (1999). Ranges of temperature (4, 15, 26, 37, and 40°C) and salinities (3.5–10%) were determined in MA incubated up to 7 days. Marine Agar was supplemented with NaCl to attain total salinities of 4, 5, 6, 7, 8, 9, and 10%. Optimal values were taken from the fastest grown plates. Specific ionic requirements were tested by assessing the growth abilities of the strain on solid media with defined combinations of four sea salts (NaCl, MgCl_2_, CaCl_2_, and KCl), according with already reported methods (Macián et al., 2005). Extracellular hydrolytic activities on casein, starch, Tween-80, and DNA were determined after 6 d incubation by using Marine Agar supplemented with 10% (v/v) casein suspension, or 0.2% (w/v) soluble starch, for the first substrates. Tween-80 Agar (Smibert and Krieg, 2007) and DNAse agar (Oxoid) were supplemented with sea salts (Marine Cation Supplement, Farmer and Hickman-Brenner, 2006). Activity on starch was revealed after lugol addition and HCl 1 N was used to show DNAse activity. Oxidase test was performed with Oxoid oxidase discs and catalase was tested with 10 vol. H_2_O_2_. API 20NE and API ZYM strips were inoculated with cell suspensions prepared in 3.5% Seasalts (Oxoid) and AUX Medium for API 20NE assimilation tubes was supplemented with a concentrated Seasalts solution to give the same salinity used in the cell suspension. Sole carbon and energy sources used for growth were tested on Basal Medium Agar as described by Baumann and Baumann (1981).

Fatty acid methyl esters were extracted from biomass grown for 48 h on MA at 26°C and prepared according to standard protocols as described for the MIDI Microbial Identification System (Sasser, 1990) at the CECT. Cellular fatty acid content was analyzed by gas chromatography with an Agilent 6850 chromatographic unit, with the MIDI Microbial Identification System using the TSBA6 method (MIDI, 2008) and identified using the Microbial Identification Sherlock software package.

## Results and discussion

### 16S rRNA gene phylogeny

Strains CECT 5113^T^, CECT 5114, CECT 5118^T^, and CECT 5120 were affiliated to the genus *Thalassobius* based on partial 16S rRNA gene sequence comparison performed among routine identification and authentication procedures at CECT. The strains had been isolated from the same samples and geographic location that rendered the type strain of *T. mediterraneus* (CECT 5383^T^ = XSM19^T^). In fact, they were provisionally identified as *T. mediterraneus* or *Thalassobius* sp. strains in the CECT catalog. The similarities of their respective 16S rRNA gene sequences to *T. mediterraneus* CECT 5383^T^ were low (only 96.6%) for strains CECT 5113^T^ and CECT 5114, which showed a closer position to *T. maritimus* (98.3%). On the other hand, strains CECT 5118^T^ and CECT 5120 showed a 99.6% similarity to *T. mediterraneus* CECT 5383^T^.

**Figure 1:**
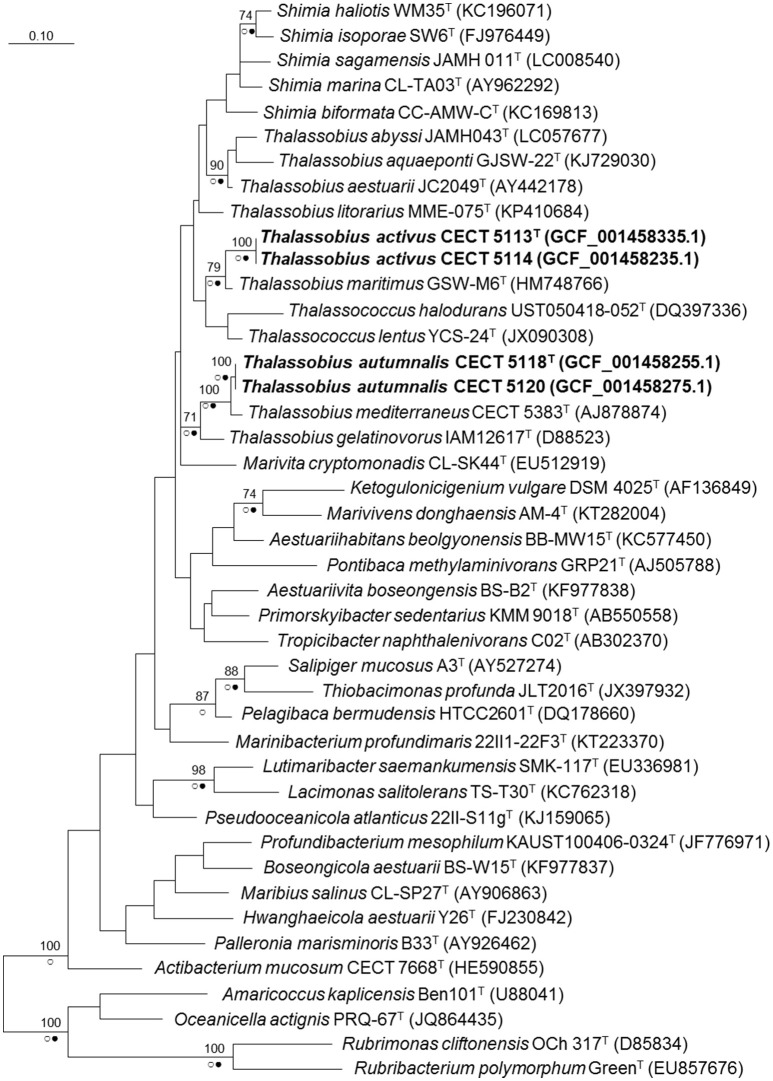
Phylogenetic reconstruction based on the 16S rRNA gene using the maximum likelihood method. Bootstrap values (if >70% and outside subclades) based on 1,000 resamplings are shown as percentages at the branch nodes. Circles indicate that corresponding nodes were recovered in trees generated with the Maximum Parsimony (open circles) or the Neighbor joining (filled circles) methods. Bar. substitutions per nucleotide position. Name of the strains corresponding to new species are indicated in bold. In parentheses, RefSeq assembly accession number.

When a phylogenetic tree based on almost complete (genome derived) 16S rRNA gene sequences was built with the whole set of *Roseobacter* group species plus these four strains (Figure 1), a clear affiliation to the genus *Thalassobius* could be seen, as the sequences are included among *Thalassobius* spp. As expected, strains CECT 5113^T^ and CECT 5114 join *T. maritimus* GSW-M6^T^ with a high bootstrap support while strains CECT 5118^T^ and CECT 5120 merge with *T. mediterraneus* CECT 5383^T^, also with high bootstrap, according with their similarity levels toward these species. The *Thalassobius* species, however, do not only include the sequences of these four strains, but also the types of the five *Shimia* species (all forming a monophyletic cluster within the *Thalassobius* group), plus the two species of *Thalassococcus*, which remain as a pair related to *T. maritimus*.

A close revision of 16S rRNA trees presented along the proposals of new species on these three genera reveal large variations in the relative positions of the taxa included in the analysis, depending on the particular set of selected taxa and the treeing method. The instability of the branching is always noticeable: for example, Nogi et al. (2015) used four *Thalassobius* spp. and no *Thalassococcus* to build their Neighbor Joining tree with the five species of *Shimia*: *Thalassobius* spp. appear as closest neighbors, forming a monophyletic cluster with the *Shimia* spp. By contrast, Hameed et al. (2013) presented a Neighbor Joining tree in which neighbors of *Shimia* are not *Thalassobius* spp., but *Nautella italica* and *Lentibacter algarum*. More examples of instability could be seen when comparing other papers describing *Roseobacter* group members. It is clear that 16S rRNA gene sequences alone are unable to resolve the phylogeny of a group displaying such complexity and overburden of genera descriptions. It seems particularly important to include not only taxa showing the highest 16S rRNA sequence similarity to the strains being considered, but a large representative set of members of the group. A more robust phylogeny is likely to be achieved by the use of whole genome information and selection of a large, optimized set of genes allowing more sound phylogenetic resolution of the clade, an approach currently in development. Genomic sequences are also valuable for the circumscription of the isolates at the species level and for providing insights into their biology, as will be reported in the coming subheadings.

### Genome sequence metrics and relatedness indices

Genome length, G+C molar content, protein and rRNA genes are summarized in Table 2 for all the genomes used. Strains CECT 5113^T^ and CECT 5114 possess genomes in the lower size and G+C range: <3.5 Mb genome size and <55 mol% G+C molar content. They are similar to *T. maritimus*, the phylogenetically closest species, which is also under 3.5 Mb genome length and was until now the one with lower G+C content (56.3 mol%). Other type strains except for *T. mediterraneus* have genomes larger than 3.9 Mb and G+C higher than 58 mol%. Strains CECT 5118^T^ and CECT 5120 had larger genomes, 4.3–4.4 Mb, and G+C molar content is among the higher in the genus, 59.8 mol%, surpassed only by the value of the *T. aestuarii* type strain (60.4 mol%).

**Table 2:** Genomic sequences employed with general features and accession numbers in public databases (an asterisk indicates those reported in this study).

**Strain**	**Mb**	**Contigs**	**G+C (mol%)**	**Protein**	**rRNA**	**tRNA**	**Accession**
*S. haliotis* DSM 28453^T^	4.0	22	58.0	4020	5	44	FOSZ01
*S. marina* CECT 7688^T^	4.0	45	57.4	3901	3	45	CYPW01
*Shimia* sp. SK013	4.0	28	57.2	3981	3	44	LAJH01
*T. activus* CECT 5113^T^	3.4	26	54.4	3316	3	41	CYTO01^*^
*T. activus* CECT 5114	3.5	26	54.5	3443	5	44	CYUE01^*^
*T. aestuarii* DSM 15283^T^	4.2	27	60.4	4135	9	51	FOTQ01
*T. autumnalis* CECT 5118^T^	4.4	48	59.8	4163	3	53	CYSB01^*^
*T. autumnalis* CECT 5120	4.4	47	59.8	4287	7	53	CYSC01^*^
*T. gelatinovorus* CECT 4357^T^	3.9	30	58.4	3800	7	47	CYSA01
*T. maritimus* DSM 28223^T^	3.3	14	56.3	3305	12	51	FQWM01
*T. mediterraneus* CECT 5383^T^	3.4	19	58.7	3299	8	47	CYSF01
*T. halodurans* DSM 26915^T^	4.0	12	58.0	3902	9	46	FNUZ01

**Figure 2:**
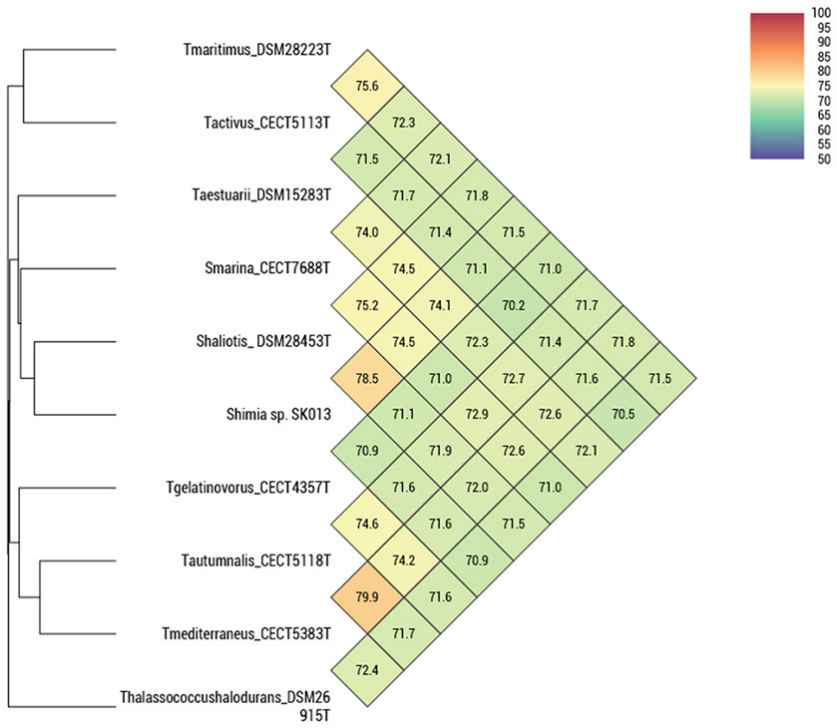
OrthoANI indices between pairs of *Thalassobius, Shimia* and *Thalassococcus* strains (according to Lee et al., 2016).

ANIb, ANIm, OrthoANI, and digital DDH were determined in order to relate the strains CECT 5113^T^, CECT 5114, CECT 5118^T^, and CECT 5120 to the eight strains of *Thalassobius, Shimia*, and *Thalassococcus* sp. with publicly available genomes. Results of these determinations are reported in Table 3 (ANIb, ANIm, digital DDH) and Figure 2 (OrthoANI).

**Table 3:** ANIb, ANIm, and estimated DDH values between genomes of *Thalassobius, Shimia*, and *Thalassococcus* species (arranged as in Figure 3).

	**1**	**2**	**3**	**4**	**5**	**6**	**7**	**8**	**9**	**10**	**11**	**12**
**ANIb**												
1 *T. maritimus* DSM 28223^T^	–											
2 *T. activus* CECT 5113^T^	75.0	–										
3 *T. activus* CECT 5114	74.8	**99.8**	–									
4 *T. aestuarii* DSM 15283^T^	71.4	70.7	70.6	–								
5 *S. marina* CECT 7688^T^	71.4	70.9	70.8	73.2	–							
6 *S. haliotis* DSM 28453^T^	71.1	70.7	70.6	73.7	74.3	–						
7 *Shimia* sp. SK013	70.8	70.3	70.2	73.2	73.4	77.8	–					
8 *T. gelatinovorus* CECT 4357^T^	70.3	69.9	69.8	71.7	70.5	70.4	70.3	–				
9 *T. autumnalis* CECT 5118^T^	71.1	70.6	70.6	71.7	72.2	71.2	70.8	73.8	–			
10 *T. autumnalis* CECT 5120	71.1	70.6	70.6	71.7	72.2	71.2	70.9	73.8	**99.9**	–		
11 *T. mediterraneus* CECT 5383^T^	71.2	71.1	71.0	71.8	71.9	71.2	70.9	73.5	79.1	79.1	–	
12 *T. halodurans* DSM 26915^T^	70.7	69.9	69.8	71.4	70.3	70.9	70.4	70.9	71.2	71.2	71.4	–
**ANIm**												
1 *T. maritimus* DSM 28223^T^	–											
2 *T. activus* CECT 5113^T^	84.1	–										
3 *T. activus* CECT 5114	84.1	**100**	–									
4 *T. aestuarii* DSM 15283^T^	84.4	84.1	83.9	–								
5 *S. marina* CECT 7688^T^	84.4	84.9	84.8	83.9	–							
6 *S. haliotis* DSM 28453^T^	83.9	84.5	84.3	83.5	85.2	–						
7 *Shimia* sp. SK013	83.6	83.9	83.8	83.4	83.9	83.8	–					
8 *T. gelatinovorus* CECT 4357^T^	82.2	84.9	85.1	83.0	83.6	82.8	82.6	–				
9 *T. autumnalis* CECT 5118^T^	84.1	84.5	84.5	83.9	85.5	83.9	83.4	83.3	–			
10 *T. autumnalis* CECT 5120	84.1	84.6	84.5	84.1	85.5	84.0	83.5	83.3	**99.9**	–		
11 *T. mediterraneus* CECT 5383^T^	84.1	85.2	85.2	84.1	84.9	83.9	83.5	83.4	85.7	85.7	–	
12 *T. halodurans* DSM 26915^T^	84.4	85.2	84.6	84.2	84.5	84.1	83.8	83.1	84.0	84.2	84.1	–
**digital DDH**												
1 *T. maritimus* DSM 28223^T^	–											
2 *T. activus* CECT 5113^T^	19.6	–										
3 *T. activus* CECT 5114	19.5	**99.9**	–									
4 *T. aestuarii* DSM 15283^T^	19.8	19.5	19.3	–								
5 *S. marina* CECT 7688^T^	21	20.7	20.6	19.6	–							
6 *S. haliotis* DSM 28453^T^	20.3	19.6	19.6	19.3	20.8	–						
7 *Shimia* sp. SK013	19.5	19.9	19.7	18.7	19.7	21.2	–					
8 *T. gelatinovorus* CECT 4357^T‘^	19.4	20.6	20.6	18.6	20.2	19.3	18.1	–				
9 *T. autumnalis* CECT 5118^T^	20.6	20.9	20.8	20.5	22.3	20.4	19.4	19.9	–			
10 *T. autumnalis* CECT 5120	20.7	21	20.9	20.6	22.2	20.6	19.5	20	**98.5**	–		
11 *T. mediterraneus* CECT 5383^T^	21.5	22.1	22	21.1	22.1	20.5	20.8	20	23.1	23.2	–	
12 *T. halodurans* DSM 26915^T^	21.5	19.9	19.6	20.2	19.2	19	19.1	18.9	20.1	20.3	20.2	–

*Values above the thresholds for species delineation are in bold*.

The four strains formed two very tight pairs related by almost 100% with all ANI calculations: strains CECT 5113^T^ and CECT 5114 were 99.8–100% related to each other and showed a maximum relatedness to the genome of the type strain of *T. maritimus* (as expected from 16S rRNA gene sequence similarity), with values of 75.0–75.1% (ANIb), 75.6–75.7% (OrthoANI), and 19.5–20.9% (digital DDH). Values with other species were even lower. According with the currently established boundaries for genomic species definition (95–96 for ANI, 70% for digital DDH) these values qualify both strains as members of a single genomic species, different from any of their closest relatives.

Strains CECT 5118^T^ and CECT 5120 also show high levels of reciprocal relatedness (99.9% ANI and 98.5% digital DDH). *T. mediterraneus*, the species most related in terms of 16S rRNA gene sequence, is also the closest, with 79.1% ANIb, 79.9% OrthoANI, and 23.2% digital DDH relatedness to them. All other *Thalassobius, Shimia*, and *Thalassococcus* species compared show even lower values. Thus, strains CECT 5118^T^ and CECT 5120 should also be considered as yet another new species from a genomic point of view.

In order to substantiate the recognition of the two novel species, a wide phenotypic characterization of the strains was undertaken, including the type strains of *T. mediterraneus, T. gelatinovorus, T. aestuarii*, and *T. maritimus*.

### Phenotypic characterization and discrimination

Strains CECT 5113^T^, CECT 5114, CECT 5118^T^, and CECT 5120 were Gram-reaction (lyse in 3% KOH) and Gram-staining negative coccobacilli or short rods. Motility was negative in all cases and previous data (Ortigosa et al., 1994) on flagella stain indicate that the strains do not synthetize flagella. All four strains grow well on Marine Agar forming circular, slightly convex colonies with entire margin, which develop in 2 days incubation at 26°C. Strain CECT 5120 was slower and grew to a density lower than the three other strains. Strain CECT 5118^T^ produced a beige to brown diffusible pigment in prolonged incubations. All strains were able to accumulate polyhydroxyalkanoates (PHA) as revealed by the Nile Red plate assay on Marine Agar plus D-glucose (Spiekermann et al., 1999).

All strains grew optimally at 26°C and with salinities ranging from that in Marine Agar (3.4% aprox.) to 5% (3.5% of mixed salts in MA plus additional 1.6% NaCl). When cultured in media of the same nutritional content as MA (1% Tryptone and 0.3% yeast extract) but deprived of salts or supplemented with 2% NaCl or 2% KCl none of the strains was able to grow, indicating a strictly halophilic nature. Strains CECT 5118^T^ and CECT 5120 were also unable to grow when the medium was supplemented with mixtures of 2% NaCl plus 0.9% MgCl_2_, 0.2% CaCl_2_, or 0.1% KCl or combinations of the four salts. Strains CECT 5113^T^ and CECT 5114 did not grow with 2% NaCl or 2% KCl or with 2% NaCl plus MgCl_2_ or CaCl_2_ separately, but were able to grow when both divalent cations, calcium, and magnesium chlorides, were added to NaCl.

The strains were poorly reactive in API ZYM and API 20NE supplemented with Marine Cations Supplement, which contains the four principal marine cations as sulfate and chloride salts. In order to better fulfill the ionic requirements of the strains, API strips were replicated using Ocean Salts (Oxoid) solution, so the final salinity was 3.5%, but reactivity on the miniaturized tests systems did not improve.

Anaerobic growth with nitrate as alternative electron-acceptor and a mixture of succinate, acetate and lactate as substrates was assayed in Baumann's denitrification medium (Baumann and Baumann, 1981) with negative results for all four strains. *T. gelatinovorus* CECT 4357^T^ was the only positive strain. Results for nitrate to nitrite reduction in API 20NE strips were in accordance with the results obtained in Baumann's medium. None of the strains was able to ferment D-glucose, a result that, in combination to the inability to live anaerobically with nitrate, usually qualifies a strain as strict aerobe.

A large number of sole carbon and energy sources for growth were tested on Baumann's Basal medium Agar supplemented with 0.2% carbohydrates, or 0.1% organic acids, amino acids, and amines. The list of used substrates is included in the species descriptions. As a general rule, organic acids were the most widely used carbon sources, although most amino acids and some carbohydrates were also used by the strains. This behavior was already described as characteristic for the genus. The spectrum of carbon sources is useful to differentiate species within the group, as indicated in Table 4.

**Table 4:** Differential characteristics between the type strains of *Thalassobius* species: 1, *T. autumnalis* sp. nov. CECT 5118^T^; 2, *T. mediterraneus* CECT 5383^T^; 3, *T. gelatinovorus* CECT 4357^T^; 4, *T. activus* sp. nov. CECT 5113^T^; 5, *T. maritimus* GSW-M6^T^ (Park et al., 2012); 6, *T. aestuarii* JC2049^T^ (Yi and Chun, 2006); 7, *T. aquaeponti* GJSW-22^T^ (Park et al., 2014); 8, *T. abyssi* JAMH043^T^ (Nogi et al., 2016); 9, *T. litorarius* MME-075^T^ (Park et al., 2016).

	**1**	**2**	**3**	**4**	**5**	**6**	**7**	**8**	**9**
Motility	–	–	+	–	+	–	–	–	–
NO${}_{3}^{-}$ reduction	–	–	+	–	+	–	+	+	–
Gelatin hydrolysis	–	–	+	–	–	+	–	–	–
Maximum temperature (°C)	37	37	40	37	30	35	35	30	35
Maximum salinity (%)	6	8	8	6	7	7	6	7	5
Use of carbon sources:									
D-cellobiose	+	–	–	+	+	+	+	+	–
Propionate	–	–	–	+	nd	nd	nd	nd	nd
L-threonine	–	–	–	+	nd	nd	nd	nd	nd
L-arginine	–	+	+	+	nd	+	nd	nd	nd
L-aspartate	–	+	+	+	nd	nd	nd	nd	nd
Fatty acid (%):									
C_10:0_ 3OH	3	2	–	7–10	5	2	2	–	tr
C_12:1_ 3OH	5	4–5	5	–	1	–	–	–	–
C_18 : 1_ *ω7c* 11 methyl	3–9	1–2	–	3–5	–	1	16	tr	13

*Unless otherwise indicated data from this study. +, positive; –, negative; nd, not determined*.

Hydrolytic extracellular activities were very scarce among *Thalassobius* strains, with two exceptions: *T. gelatinovorus* CECT 4357^T^ and *T. aestuarii* CECT 8650^T^ were able to degrade gelatin, but other strains did not show hydrolysis on any of the substrates tested: gelatin, casein, Tween-80, starch, or DNA.

Cellular fatty acid composition was determined for the eight strains alongside with the result reflected in Table 5. The main fatty acid for all strains is included in the Summed Feature 8, corresponding to C_18 : 1_ ω*7c*/C_18 : 1_ ω*6c*, which accounts for 72–88% of the total. It is also the dominant fatty acid in the whole *Rhodobacteraceae* family. The second most abundant, common fatty acid, is C_16 : 0_ (3–9%) while C_18 : 0_ is also common to all species but accounts for 1–2.6%. The rest of fatty acids have a differential distribution among species, with C_18 : 1_ ω*7c* 11methyl, C_10 : 0_ 3OH, and C_12 : 1_ 2OH amounting up to 9% in some species.

**Table 5:** Fatty acid composition of 1, *T. activus* CECT 5113^T^; 2, *T. activus* CECT 5114; 3, *T. autumnalis* CECT 5118^T^; 4, *T. autumnalis* CECT 5120; 5, *T. gelatinovorus* CECT 4357^T^; 6, *T. mediterraneus* CECT 5383^T^; 7, *T. maritimus* CECT 8650^T^; 8, *T. aestuarii* CECT 8650^T^.

	**1**	**2**	**3**	**4**	**5**	**6**	**7**	**8**
**HYDROXYLATED**								
C_10:0_ 3OH	7.6	9.7	2.5	2.7		2.2	5.5	2.3
C_12:1_ 3OH			5.1	4.9	5.2	4.6	1.2	
C_16:0_ 2OH			tr		1.2			1.5
C_17:0_ iso 3OH				2.6			2.7	2.4
**SATURATED**								
C_12:0_			tr		tr			
C_16:0_	9.3	9.2	4.1	3.8	4.7	4.7	6.3	3.0
C_17:0_					2.8	tr	tr	tr
C_18 : 0_	1.2	2.1	1.8	1.8	1.8	2.3	2.6	1.0
**UNSATURATED**								
Summed Feature 3	tr	tr	tr	tr		1.0		
Summed Feature 8	78.3	73.6	81.0	72.4	80.6	82.2	80.9	88.2
C_18 : 1_ *ω7c* 11 methyl	3.0	4.8	3.5	9.5		1.4		1.0
C_18 : 1_ *ω9c*			tr	tr	1.4	tr		
C_20:1_ *ω7c*			tr	1.3				

*Summed Feature 3: C_16:1_ ω7c/ω6c; Summed Feature 8: C_18 : 1_ ω7c/ω6c. All values determined in this study*.

Differentiation among species of the genus based on experimental phenotypic tests could be achieved as indicated in Table 4.

### Genome derived features of strains CECT 5113^T^, CECT 5114, CECT 5118^T^, and CECT 5120

After annotation with RAST and Prokka, using the SEED Viewer (Overbeek et al., 2014) and BIOiPLUG to explore annotated genomes and Metacyc as a resource for metabolic pathway information (Caspi et al., 2016), the following features could be predicted from genome sequences:

1. Chemotaxonomic traits: A set of genes (*cdsA, pgsA, pgpA, pgpB*, and *cls*) coding for polar lipid biosynthetic enzymes for phosphatidylglycerol (EC 2.7.7.41, EC 2.7.8.5, and EC 3.1.3.27) and for phosphatidyl choline (EC 2.7.8.24) production was present but those for phosphatidyl ethanolamine, sulfoquinovosyl diacyl glycerol, diphosphatidyl glycerol (cardiolipin), phosphatidyl serine, or phosphatidyl *myo* inositol biosynthetic enzymes were not found. The presence of the gene coding for enzyme decaprenyl diphosphate synthase (EC 2.5.1.91) suggests ubiquinone 10 as major isoprenoid quinone. Polyamine biosynthetic abilities comprise, at least, production of putrescine, via arginine decarboxylase (*speA*) plus agmatinase (*speB*). Strains CECT 5113^T^ and CECT 5114 encode carboxy *nor*-spermidine dehydrogenase and carboxy *nor*-spermidine decarboxylase, but genes for these enzymes are absent from strains CECT 5118^T^ and CECT 5120. Peptidoglycan composition: pentapeptide was predicted to contain *meso*-DAP in third position, as predicted by the presence of *murE*, coding for UDP-N-acetyl muramoyl alanyl D-glutamate- 2,6 diaminopimelate ligase (EC 6.3.2.13).
2. Polyhydroxyalkanoate accumulation and degradation: the four strains present a complete set of genes for this subsystem.
3. Motility: No gene related to flagellar synthesis and movement was annotated in the genomes of strains CECT 5118^T^ and CECT 5120, in full agreement with their non-motile behavior. Strains CECT 5113^T^ and CECT 5114, also non motile, contain up to 66 genes in the subsystem, but apparently lack some essential ones (as the flagellin gene), among others, according to RAST annotation. However, in the Prokka-annotated version of these genomes flagellin was detected by BIOiPLUG search.
4. Cell cycle: the absence of the *minCDE* in the cytoskeleton set of genes is notorious; this absence is, to the best of our knowledge, common to the *Roseobacter* group and it might be related to the uneven division behavior found in several *Rhodobacteraceae* taxa. In addition, strains CECT 5113^T^ and CECT 5114 lack *ftsQ* and strains CECT 5118^T^ and CECT 5120 lack *mreD*.
5. DNA metabolism and Horizontal Gene Transfer-related functions. None of the four strains contains a CRISPR/Cas system but defense against foreign DNA in the form of a restriction-modification system type I is present in the four strains. An additional type III R-M system is present in strain CECT 5118. All four strains contain a Gene Transfer Agent (GTA) set of genes. This GTA is universal in the *Rhodobacteraceae* so far explored, but the members of *Thalassobius, Shimia*, and *Thalassococcus* genera all lack the major capsid protein gene. Other horizontal transfer mechanisms seem to be present in strains CECT 5118^T^ and CECT 5120: they have the machinery to produce a type IV secretion system (T4SS) which may mediate protein and nucleoprotein transfer. Strains CECT 5113^T^ and CECT 5114 lack T4SS. Plasmid related functions were not predicted by RAST search, but all the four strains contain the plasmid replication genes *repA, repB*, and *repC*, characteristic of repABC type plasmids of *Rhodobacteraceae* that may also contain the T4SS (Petersen and Wagner-Döbler, 2017).
6. Cell to cell communication and adherence: Although none of the strains presents identifiable quorum-sensing systems, they all show the two component *chv* regulatory system characteristic of *Alphaproteobacteria*, involved in the regulation of exopolysaccharide synthesis and symbiotic recognition in *Rhizobium*. It is also noticeable the presence in the four strains (and in the rest of *Thalassobius* type strains) of a T2SS related to tight adherence (*tad*) gene cluster, involved in tenacious biofilm formation, as well as several genes encoding for capsular exopolysaccharide synthesis.
7. Stress response: Osmotic stress-related genes code for a complete betaine synthesis from choline. Oxidative stress is counteracted by the presence of catalase, peroxidase and a Fe-containing superoxide dismutase (SOD).
8. Light-related genes: None of the strains contains genes for the synthesis of a photosynthetic apparatus, but *regA*, a part of a two-component system related to the expression of photosynthesis in anaerobiosis, is predicted in all four strains; *hvrA*, a transcriptional activator of *puf* and *puh* genes at dim light intensities, is found only in strains CECT 5118^T^ and CECT 5120. All four lack genes for rhodopsin synthesis.
9. Anaerobic respiration. Nitrate reduction and denitrification: the strains do not possess respiratory nitrate reductase, in agreement with the negative results obtained in the corresponding test. They lack also the nitrous oxide and nitric oxide reductases needed for denitrification. Dimethyl sulfoxide (DMSO) reductase and thiosulfate or sulfite reductases are also absent but an arsenate reductase gene is detected in all strains.
10. Sulfur metabolism: Sulfur oxidation is predicted from the genomes of strains CECT 5118^T^ and CECT 5120 (a behavior common to several taxa in the *Rhodobacteraceae*), but not from the genomes of strains CECT 5113^T^ and CECT 5114, due to the lack of several *sox* genes (*soxA, X, W*, and *H)*. Sulfite oxidase is absent in all four. Another well studied, widely represented activity on sulfur compounds, the degradation of dimethyl sulfoniopropionate (DMSP), is present in the genomes of strains CECT 5118^T^ and CECT 5120 with a complete demethylation pathway. Strains CECT 5113^T^ and CECT 5114 present *dmdA* (demethylase) but it is uncertain if the pathway is complete (apparently, they lack the 3-methylmercapto propionyl-CoA ligase). On the other hand, strain CECT 5114 possesses a gene for DMSP lyase, thus being able to degrade DMSP directly to dimethylsulfide (DMS) plus acrylate.
11. Carbon monoxide oxidation: together with sulfur oxidation, this trait is present in several members of the *Roseobacter* group and apparently functions as a complementary energy resource. Aerobic carbon monoxide dehydrogenase is present in the genomes of strains CECT 5118^T^ and CECT 5120, but not in strains CECT 5113^T^ or CECT 5114.
12. Membrane transport (solutes): strains CECT 5118^T^ and CECT 5120 possess the highest number of genes involved in ABC transporters (86) while strains CECT 5113^T^ and CECT 5114 contain only 35–43 genes in this category. TRAP transporters account for 31–32 genes on strains CECT 5118^T^ and CECT 5120 and 32–36 in strains CECT 5113^T^ and CECT 5114.
13. Central metabolism and respiration: the four strains have a complete tricarboxylic acid cycle and aerobic respiration. The ethyl malonyl-CoA pathway for C2 assimilation is also complete in all four strains. Glycolytic abilities, on the other hand, include incomplete glycolysis (no phosphofructokinase is predicted), nor Entner-Doudoroff or Pentose phosphate pathways for strains CECT 5118^T^ and CECT 5120. Strain CECT 5113^T^ has a complete pentose phosphate and incomplete glycolytic and KDPG routes while strain CECT 5114 has incomplete versions of Pentose Phosphate and KDPG pathways. In any case, the four strains manage to use and grow with several carbohydrates, including D-ribose, some hexoses, a few disaccharides (as lactose, all present β-galactosidase activity) and some uronic acids. The precise combination of carbohydrates being used by the strains in experimental tests is useful for the species differentiation (Table 4).
14. CO_2_ fixation is not predicted for any of the four strains.
15. Metabolism of aromatic compounds: the strains do not possess genes encoding for complete pathways for aromatic ring cleavage nor they encode dioxygenases, whereas some members of the genus do.

Selected genome-predicted differences for these two pairs and other *Thalassobius, Shimia*, and one *Thalassococcus* spp. are summarized in Table 6.

**Table 6:** Selected differential characteristics between the genomes of *Thalassobius, Shimia*, and *Thalassococcus* species: 1, *T. autumnalis* sp. nov. CECT 5118^T^ and CECT 5120; 2, *T. mediterraneus* CECT 5383^T^; 3, *T. gelatinovorus* CECT 4357^T^; 4, *T. activus* sp. nov. CECT 5113^T^ and CECT 5114; 5, *T. maritimus* DSM 28223^T^; 6, *T. aestuarii* DSM 15283^T^; 7, *S. marina* CECT 7688^T^; 8, *S. haliotis* DSM 28453^T^; 9, *T. halodurans* DSM 26915^T^.

	**1**	**2**	**3**	**4**	**5**	**6**	**7**	**8**	**9**
Entner-Doudoroff pathway, classical	–	–	+	–	+	+	–	+	+
α- and β-galactosidades	+	–	–	+	+	–	+^‡^	+	+
Carbon monoxide oxidation (CODH)	+	+	+	–	–	+	+	+	+
CO_2_ fixation, Calvin cycle									
RuBisCo	–	–	+	–	+	–	–	–	–
Phosphoribulokinase	–	–	+	–	–	–	–	–	–
Protocatechuate dioxygenase and 3,4 dihydroxyphenylacetate 2,3 dioxygenase	–	–	+	–	–	+	–	–	+
Denitrification	–	–	+^*^	–	+	+^§^	–	+^*^	–
Aryl sulfatase	–	–	–	–	–	+	+	+	–
DMSP lyase (DMSP to DMS + acrylate)	–	–	–	v	–	+	–	+	+
*Sox* genes (sulfur oxidation)	+	–	+	–^†^	–	+	–	–	+
*HvrA* (transcriptional activator for *puf/puh*)	+	+	+	–	–	+	+	+	+
Type IV secretion system	+	+	–	–	–	–	+	–	+
Restriction-Modification systems	I, III	–	I	I	–	I	I	I	–
Gene Transfer Agent, GTA (no Capsid gene)	+	–	+	+	+	+	+	+	+
*RepABC* plasmid replication module	+	+	–	+	+	+	+	–	+
Multidrug efflux pumps	+	+	+	–	+	+	+	+	–
Phospholipids	PG, PC	PG, PC	PG, PC	PG, PC	PG, PC	PG, PC, DPG	PG, PC, DPG	PG, PC, DPG	PG, PC, PE
Polyamine biosynthetic enzymes:									
Carboxy *nor*-spermidine	–	+	+	+	+	+	+	–	+
dehydrogenase and decarboxylase								–	
G+C (mol%)	59.8	58.7	58.4	54.4	56.3	60.4	57.4	58.0	58.0

* *Only N_2_O reduction pathway*.

§ *Only NO and N_2_O reduction pathway*.

† *soxA, X, W and H, missing*.

‡ *Only β-galactosidase. +, positive, –, negative, v, variable between strains*.

Considering both the experimentally determined features and the presence of genes predicting given phenotypic traits in the genomes, there are enough differences between strains and their closely related species for unequivocally differentiate the genomic groups previously delineated (Tables 4, 6). Genomic, phylogenetic and phenotypic information thus confirm that the four strains represent two novel species. They fulfill the basic characteristics defining the genus *Thalassobius* (Arahal et al., 2005) and have *Thalassobius* species as their closest phylogenetic neighbors in both cases, thus, they should be recognized as new *Thalassobius* species: we propose the name of *Thalassobius activus*, for strains CECT 5113^T^ and CECT 5114, with CECT 5113^T^ as type strain. Strains CECT 5118^T^ and CECT 5120 constitute another new species, for which we propose the name of *Thalassobius autumnalis*, with CECT 5118^T^ as type strain. Protologues for both species are included at the end of the section.

### Phylogenomic analysis of genera *thalassobius, shimia*, and *thalassococcus*

**Figure 3:**
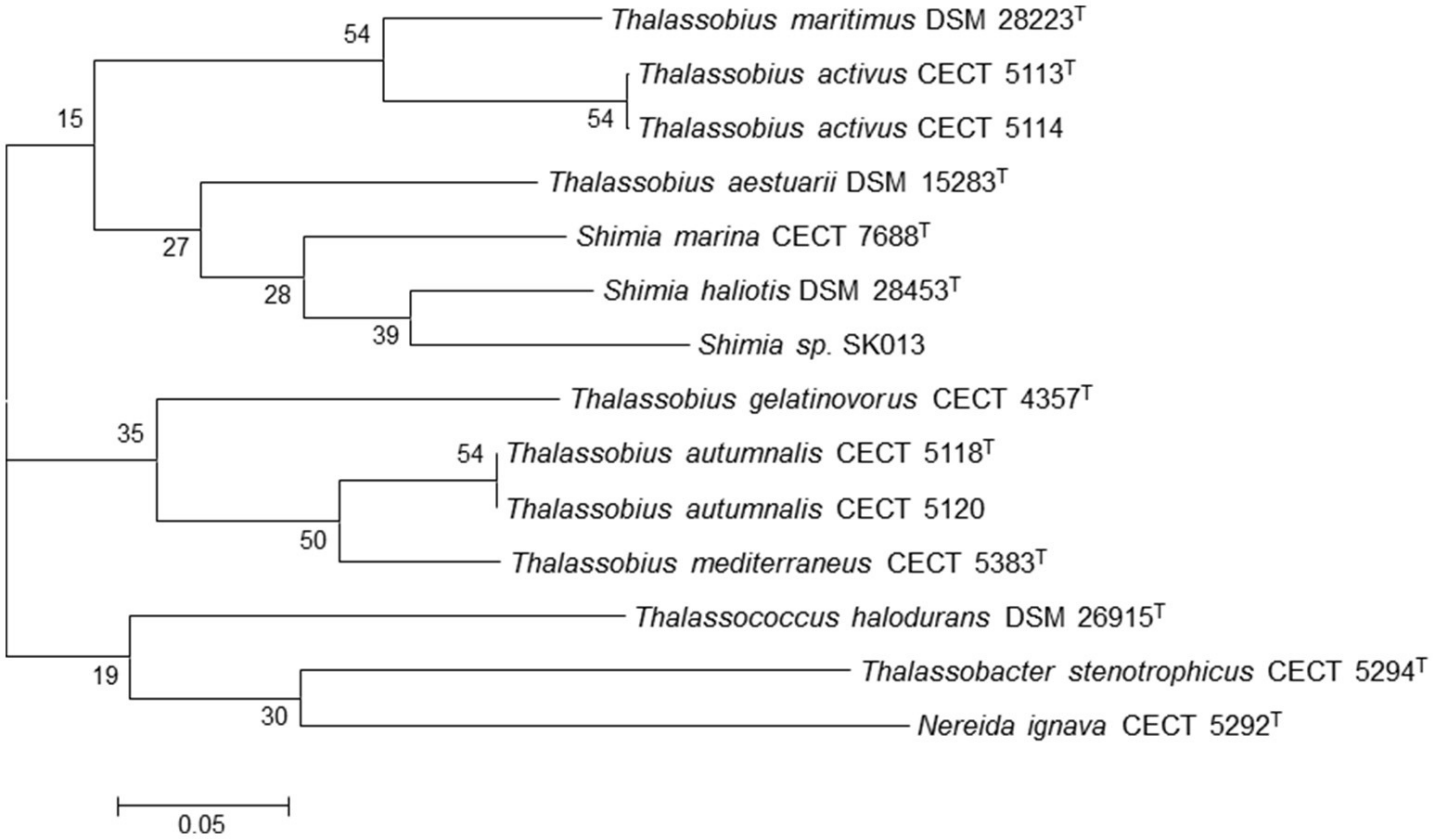
Phylogenetic tree generated with BCG54. *Thalassobacter stenotrophicus* CECT 5294^T^ (CYRX01) and *Nereida ignava* CECT 5292^T^ (CVQV01) were included as outgroup organisms. The numbers at the nodes indicate the gene support index (maximal value is 54).

As previously pointed, another goal of this study was to study the relationships between the species of the genera *Thalassobius, Shimia*, and *Thalassococcus* by using genome-derived data. All publicly available type strain genomes of species in these three genera have been considered together with the genome from an unclassified *Shimia* species (Kanukollu et al., 2016) that has been proven to constitute a separate genomic species (Tables 2, 3). The tree generated with BCG54 using *Thalassobacter stenotrophicus* CECT 5294^T^ (CYRX01) and *Nereida ignava* CECT 5292^T^ (CVQV01) as outgroup is shown in Figure 3. The tree confirms the phylogenetic relationships previously suggested by 16S rRNA gene sequence comparison and genomic relatedness, confirming the close position of *T. activus* to *T. maritimus* and of *T. autumnalis* to *T. mediterraneus*. The grouping of *T. mediterraneus* plus *T. autumnalis* with *T. gelatinovorus*, a regular finding with other approaches, is also confirmed. All three *Shimia* species appear inside the *Thalassobius* genus, as closest relatives of *T. aestuarii*, while *T. halodurans* separates from *Thalassobius* and *Shimia*, and merges with the pair used as an outgroup, discarding the topology that *Thalassococcus* spp. occupied in the 16S rRNA tree. The results obtained from the BCG54 tree do not sustain monophyly for *Thalassobius* as currently defined. Furthermore, monophyly for *Thalassobius*—*Shimia* grouping is not resolved, as the group formed by *T. mediterraneus-T. autumnalis-T. gelatinovorus* does not merge with the rest of species before the last node. A better resolution of this node might be attained if the genomes of all *Shimia, Thalassobius*, and *Thalassococcus* were available, but, for the moment, we only can conclude that there are three possible monophyletic groups to be considered: the core *Thalassobius* spp. or *Thalassobius* spp. *sensu stricto* (containing the type species *T. mediterraneus* plus *T. autumnalis* and *T. gelatinovorus*), the one of *T. maritimus* plus *T. activus* sp. nov. and the one of *Shimia* spp. plus *T. aestuarii*. A strict taxonomic procedure would split *Thalassobius* spp. in three genera, with *T. aestuarii* (and perhaps others, as *T. abyssi, T. aquaeponti*, and *T. litoreus*) being reclassified as new combinations of *Shimia*, and *T. maritimus* plus *T. activus* being proposed as a new genus. But as already commented, this would be premature, since the addition of more type strain genomes would eventually reveal if the whole group constitutes a monophyletic lineage at higher levels, thus allowing a reclassification of all *Shimia* spp as *Thalassobius* new combinations, a solution that, in our opinion, would be also possible and more clarifying than the splitting option, which would imply to describe more and more indistinguishable genera within the *Roseobacter* group.

### Genome derived features of *thalassobius* spp., *shimia* spp., and *thalassococcus halodurans*: common and differential features

A survey of the gene content of the *Thalassobius* spp., *Shimia* spp., and *T. halodurans* draft genomes was conducted in order to enlighten the taxonomic status of the three genera. We looked for traits especially used for taxonomic work at the genus level. Genomic traits that are differential among species and/or genera are presented in Table 6. A list of features shared by all three genera representatives is included as Table 7.

**Table 7:** A list of common genomic features shared by *Thalassobius-Shimia* group included in the study.

- A complete set of PHB synthesis and degradation machinery
- Type II secretion system, tad type
- *chv* two components response regulator
- Absence of *BChl a* production, together with presence of *regA* gene
- Absence of proteorhodopsin production
- Presence of a Genome Transfer Agent set (GTA) lacking Capsid gene
- Lack of *minCDE* system among cytoskeleton set of genes
- Putrescine biosynthetic ability (arginine decarboxylase, *speA*, plus agmatinase*, speB*)
- Phosphatidyl choline synthesis (*pcs*)
- Decaprenyl phosphate synthase (Q10)
- A complete ethylmalonyl CoA pathway for C2 assimilation
- Arsenate reductase
- Mercuric reductase

All three genera representatives share characteristics owned by the whole *Rhodobacteraceae* family, as the peptidoglycan composition (with *m*-DAP at 3rd position), predominant ubiquinone (Q10), major cellular fatty acid (C_18 : 1_ ω*7c*/C_18 : 1_ ω*6c*) and a G+C molar content over 50%. Maybe, the lack of *minCDE* genes should be added to this list of traits common to all *Rhodobacteraceae*: the absence of these genes in various *Rhodobacteraceae* was already noted by Lutkenhaus (2007) and we have been unable, so far, to find any member of this group that contained this part of the cytoskeletal machinery. Obviously, these shared common traits (symplesiomorphies) should not be considered as relevant to back a genera fusion proposal. But there are other that are shared by these particular genera (synapomorphies): we would highlight, for example, the GTA gene set composition, characterized by the common absence of capsid gene, which seems to be exclusive of these genera in contrast to the other >40 genomes of *Roseobacter* group members that could be compared in the Seed Viewer. The ability to accumulate polyhydroxyalkanoates, although not exclusive, would be another common character, not shared with all other *Rhodobacteraceae*. Other candidate traits are indicated in Table 7.

On the other hand, several genomic differences were found that allow species discrimination, as well as differentiation of the three phylogenetic clusters revealed by gene and multigenic trees: polar lipid synthesis, for example, pull apart the *T. aestuarii-Shimia* spp. group by the exclusive presence of DPG synthetic ability, while *Thalassococcus* could be differentiated by the unique presence of gene *psd*, encoding a phosphatidyl ethanolamine-synthetizing enzyme, phosphatidyl serine decarboxylase (EC 4.1.1.65), which is absent from all other species. The absence of genes encoding for PE biosynthesis in all *Thalassobius* species was surprising, as Park et al. (2012) reported the detection of PE in the type strains of four species (*T. maritimus, T. aestuarii, T. gelatinovorus*, and *T. mediterraneus*) by using TLC chromatography. But genomes of these type strains did not contain phosphatidylserine decarboxylase gene and PE synthesis is not predicted by RAST for any of the eight *Thalassobius* strains whose genomes have been explored. Genes for key enzymes of the alternative routes for PE synthesis, ethanolamine phosphotransferase (EC 2.7.8.1) or L-serine phosphatidylethanolamine phosphatidyl transferase (EC 2.7.8.29) were also undetected. Undoubtedly, this is a point that needs more attention, because of the importance that is given to polar lipid composition in bacterial taxonomy.

Absence of carbon monoxide dehydrogenase and *hvrA* gene are characteristic of *T. maritimus-T. activus*, while, the presence of aryl sulfatase is exclusive of the *Shimia* spp.*-T. aestuarii* group.

It is interesting to highlight that *T. aestuarii* shares with *T. gelatinovorus* the ability to degrade aromatic compounds, as confirmed through Aromadeg (Duarte et al., 2014) showing a complete that both type strains contain genes coding for extradiol dioxygenases of Viccinal Chelate superfamily and Lig B superfamily (for degrading monocyclic substrates in the first case and homoprorocatechuate and protocatechuate, in the second) and Rieske non-heme iron oxygenases of the phthalate dioxygenases family. However, only *T. aestuarii* DSM 15283^T^ genome harbors an extradiol dioxygenase of the Cupin superfamily, which anables to degrade gentisate and, on the other side, *T. gelatinovorus* CECT 4357^T^ possesses an additional phthalate oxygenase and a benzoate oxygenase. *T. halodurans* DSM 26915^T^ genome also encodes two extradiol dioxygenases of Viccinal Chelate superfamily and one Rieske non-heme iron oxygenases of the phthalate dioxygenases. The three *Shimia* genomes were also checked with Aromadeg (default parameters) but no protein had matches with the database.

In a recent phylogenomic study, Simon et al. (2017) analyse the evolutionary adaptation of *Rhodobacteraceae* to marine and non-marine habitats. Among other things, they conclude that the so-called *Roseobacter* group is not monophyletic, but their members derived from a common marine ancestor shared with other non-marine, non-halophilic *Rhodobacteraceae* (*Rhodobacter* and *Paracoccus*, mainly), the later representing an adaptation to non-marine habitats. A selection of genome-predicted, habitat-correlated enzymes is shown in their Table 1, including several lost in non-marine habitats plus others that were gained in marine habitats. The analysis of Simon et al. (2017) included no *Thalassobius, Shimia*, or *Thalassococcus* (the genome labeled as “*Thalassiobium* sp. R2A62” is completely unrelated to any true *Thalassobius*), so we have explored the distribution of the marine-related enzymes they reported in the genomes employed in this paper, as all of them pertain to marine inhabitants. Results are indicated in Table 8. As it can readily be observed, the five enzymes classified under the category of “Lost in non-marine habitats” are all present in the genomes of *Thalassobius* spp. (6 species), *Shimia* spp (3 species), and in the single *Thalassococcus* species included. The only significant exception was the genes for large, medium, and small carbon monoxide dehydrogenase chains, absent from the genomes of *T. maritimus* and *T. activus* sp. nov. (both strains), a trait already highlighted as characteristic of this small clade (Figure 3). On the other hand, enzymes apparently “gained in marine habitats” are not widely represented in *Thalassobius* or *Shimia* genomes. A few interesting exceptions are the betaine homocysteine S-methyl transferase (EC 2.1.1.5), involved in glycine-betaine catabolism, present in all these genomes, and the nitrile hydratase (EC 4.2.1.84), absent only from the genomes *T. activus* sp. nov. and *T. halodurans*, but present in the rest. Two additional occurrences are worth to note: γ-butyrobetaine dioxygenase (EC 1.14.11.1), participating in carnitine biosynthesis, is found exclusively in the genomes of *Thalassobius* spp. *sensu stricto* (*T. mediterraneus, T. autumnalis* sp. nov., and *T. gelatinovorus*) while aryl sulfatase (EC 3.1.6.1) is present only in *T. aestuarii* and *Shimia* spp. (as already mentioned); thus, these two enzymes could be used as discriminant traits to differentiate the respective groups.

**Table 8:** Habitat-related enzymes and their presence in the genomes of different *Thalassobius, Shimia* and *Thalassococcus* type and reference strains: 1, *T. autumnalis* sp. nov. CECT 5118^T^ and CECT 5120; 2, *T. mediterraneus* CECT 5383^T^; 3, *T. gelatinovorus* CECT 4357^T^; 4, *T. activus* sp. nov. CECT 5113^T^ and CECT 5114; 5, *T. maritimus* DSM 28223^T^; 6, *T. aestuarii* DSM 15283^T^; 7, *S. marina* CECT 7688^T^; 8, *S. haliotis* DSM 28453^T^; 9, *T. halodurans* DSM 26915^T^.

**Enzyme**	**EC number**	**1**	**2**	**3**	**4**	**5**	**6**	**7**	**8**	**9**
Gained in marine habitats^a^										
Ectoine synthase	4.2.1.108	–	–	–	–	–	–	–	–	–
Betaine homocysteine S-methyl transferase	2.1.1.5	+	+	+	+	+	+	+	+	+
γ-butyrobetaine dioxygenase	1.14.11.1	+	+	+	–	–	–	–	–	–
TMA^b^ corrinoid protein Co-methyl transferase	2.1.1.250	–	–	–	–	–	–	–	–	–
TMA^b^ N-oxyde reductase	1.6.6.9	–	–	–	–	–	–	–	–	–
Nitrile hydratase	4.2.1.84	+	+	+	–	+	+	+	+	–
Aryl sulfatase	3.1.6.1	–	–	–	–	–	+	+	+	–
Precorrin 3B synthase	1.14.13.83	–	–	–	–	–	–	–	–	–
Lost in non-marine habitats^a^										
(S)-2-haloacid dehalogenase	3.8.1.2	+	+	+	+	+	+	+	+	+
Mercury (Hg) II reductase	1.16.1.1	+	+	+	+	+	+	+	+	+
Carbon monoxide dehydrogenase	1.2.99.2	+	+	+	–	–	+	+	+	+
Precorrin-8X methylmutase	5.4.1.2	+	+	+	+	+	+	+	+	+
Precorrin-4 C11 methyl transferase	2.1.1.133	+	+	+	+	+	+	–	+	+

a *According to Simon et al. (2017)*.

b *TMA, trimethylamine. +, present; –, not detected in annotation*.

So, this analysis proves there is room for either a proposal of reunification of (at least) *Shimia* spp. with *Thalassobius* spp. under the name of *Thalassobius*, but also for the splitting of *Thalassobius-Shimia* in three genera (one of them a new one for *T. maritimus-T. activus*). However, any decision should be postponed until the gap in genomes of type strains pertaining to the group is closed.

## Conclusions

This study provides the first taxogenomic approach conducted on the genus *Thalassobius* and permits the classification of four isolates into two novel species, whose description is given below. An effort has been done to explore the possibilities of phenotypic inference opening a trend for similar studies. It also provides an analysis of the complexity for classification at the genus level within the *Roseobacter* group, here illustrated by the case of the genera *Thalassobius-Shimia-Thalassococcus*.

### Description of *thalassobius activus* sp. nov.

*Thalassobius activus* (ac.ti'vus. L. masc. adj. *activus*, active, referring to the metabolic activity of the type strain).

Cells are Gram-reaction-negative, non- motile coccobacilli. Aerobic and chemoorganotroph, they grow on Marine Agar as regular unpigmented circular colonies. Cells accumulate PHB. Mesophilic, able to grow from 15 to 26°C (optimum, 26°C), but not a 4 or 37°C. Slightly halophilic, requires sodium, calcium and magnesium salts for growth. Maximum salinity for growth is 6% (optimum 3.5–5%), no growth is obtained without salt, at 7% or above. Oxidase and catalase are positive. Fermentation of carbohydrates, nitrate reduction and denitrification are negative. Negative for urea, casein, gelatin, starch, Tween-80 and DNA hydrolysis. Indole production from tryptophan and arginine dihydrolase are negative, PNPG and alkaline phosphatase are positive.

Carbon sources used for growth included D-cellobiose, sucrose, D-melibiose, D-mannitol, *m*-inositol, acetate, pyruvate, propionate, butyrate, *t*-aconitate, citrate, 2-oxoglutarate, succinate, lactate, 3-hydroxybutyrate, glycine, L-leucine, L-serine, L-threonine, L-arginine, L-glutamate, L-alanine, L-tyrosine, L-ornithine, 4-aminobutyrate, and L-aspartate. No growth is obtained with D-ribose, L-arabinose, D-xylose, D-glucose, D-fructose, D-galactose, D-trehalose, D-mannose, L-rhamnose, maltose, lactose, salicin, amygdalin, D-glycerol, D-sorbitol, D-gluconate, D-glucuronate, D-galacturonate, D-glycerate, D-saccharate, L-citrulline, L-histidine, L-lysine, L-sarcosine, or putrescine.

Major cellular fatty acids are C_18 : 1_ ω*7c*/ω*6c* and C_10 : 0_ 3OH.

The predominant respiratory quinone, Q10 was inferred from annotated gene encoding decaprenyldiphosphate synthase (EC 2.5.1.91). Major polar lipids, PG and PC, were inferred from annotated genes encoding CTP-phosphatidate cytidyl transferase (EC 2.7.7.41), phosphatidyl glycerophosphate synthase (EC 2.7.8.5), phosphatidyl glycerophosphatase (EC 3.1.3.27), and phosphatidyl choline synthase (EC 2.7.8.24), in the genomes of strains CECT 5113^T^ and CECT 5114.

The G+C content of the DNA is 54.4–54.5 mol% (partial genome sequences).

The type strain, CECT 5113^T^ (=11SM13^T^ =LMG 29900^T^) was isolated from coastal seawater, Mediterranean Sea, at Vinaroz coast, Spain.

### Description of *thalassobius autumnalis* sp. nov.

*Thalassobius autumnalis* (au.tum.na'lis. L. adj. *autumnus*, fall, autumn, after the season of isolation).

Cells are Gram-reaction negative, non-motile coccobacilli, aerobic, and chemoorganotroph. Grows on Marine Agar forming regular circular colonies. Some strains produce a brown diffusible pigment on prolonged incubation. Polyhydroxybutyrate (PHB) is accumulated in the cells. Mesophilic and slightly halophilic, growth is observed from 15 to 37°C (optimum, 26°C), but not at 4 or 40°C and up to 6% total salinity in media supplemented with sea salts (optimum 3.5–5%). Does not grow at 7% or more salinity. It displays complex ionic requirements, as it is unable to grow either without added salts or with addition of simple salts (NaCl, KCl, MgCl_2_, CaCl_2_). Oxidase and catalase positive. Nitrate reduction to nitrite or N_2_ is negative. Does not ferment carbohydrates. Negative for urea, casein, gelatin, starch, Tween-80, and DNA hydrolysis. Indole production from tryptophan and arginine dihydrolase are negative, PNPG (β-galactosidase) and leucine arylamidase are positive.

The following sole carbon and energy sources are used for growth: D-ribose, D-xylose, D-glucose, D-mannose, D-galactose, D-cellobiose, sucrose, salicin, N-acetyl-D-glucosamine, D-glycerol, *m*-inositol, acetate, pyruvate, propionate, butyrate, *t*-aconitate, citrate, 2-oxoglutarate, succinate, malate, lactate, 3-hydroxybutyrate, L-leucine, L-serine, L-glutamate, L-alanine, L-tyrosine, L-ornithine, 4-aminobutyrate, L-sarcosine, and putrescine. Growth is negative with L-arabinose, D-fructose, D-trehalose, L-rhamnose, maltose, lactose, D-melibiose, D-amygdalin, D-mannitol, D-sorbitol, D-gluconate, D-glucuronate, D-galacturonate, D-glycerate, D-saccharate, glycine, L-threonine, L-arginine, L-citrulline, L-histidine, L-lysine, and L-aspartate.

Major cellular fatty acids are C_18 : 1_ ω*7c*/ω*6c* and C_18 : 1_ ω*7c* 11-methyl.

The predominant respiratory quinone, Q10 was inferred from annotated gene encoding decaprenyldiphosphate synthase (EC 2.5.1.91). Major polar lipids, PG and PC, were inferred from annotated genes encoding CTP-phosphatidate cytidyl transferase (EC 2.7.7.41), phosphatidyl glycerophosphate synthase (EC 2.7.8.5), phosphatidyl glycerophosphatase (EC 3.1.3.27), and phosphatidyl choline synthase (EC 2.7.8.24), in the genomes of strains CECT 5118^T^ and CECT 5120.

The G+C content of the DNA is 59.8 mol% (partial genome sequences).

The type strain, CECT 5118^T^ (=XSM11^T^ =LMG 29904^T^) was isolated from coastal seawater, Mediterranean Sea, at Vinaroz coast, Spain.

## Author contributions

DA and MP: Designed the study; LR-T: Obtained the genomes and did the mainstream processing; TL, LR-T, and MP: Further analyzed the annotations; MP: Drafted the manuscript and conducted the phenotypic testing; TL and DA: Did the phylogenomic analysis; all authors corrected and approved the manuscript.

### Conflict of interest statement

The authors declare that the research was conducted in the absence of any commercial or financial relationships that could be construed as a potential conflict of interest.
